# Acute lupus pneumonitis resembling miliary tuberculosis: A case-based review

**DOI:** 10.1515/biol-2022-0751

**Published:** 2023-12-31

**Authors:** Xin Li, Yuan Wang, Baoyu Zhang, Xiaoxia Jia, Lin Mu, Jing Ke

**Affiliations:** Center for Endocrine Metabolism and Immune Diseases, Beijing Luhe Hospital, Capital Medical University, Beijing, 101149, China

**Keywords:** systemic lupus erythematosus, acute lupus pneumonitis, miliary tuberculosis

## Abstract

Systemic lupus erythematosus (SLE) is an autoimmune disease involving multiple systems and organs, with pulmonary involvement known to be associated with disease prognosis and mortality. Acute lupus pneumonitis (ALP) resembling miliary tuberculosis (TB) is rare. Here, we present a case of ALP mimicking miliary TB and review the literature. A 19-year-old male student was referred to our hospital with fever. Although the patient met the diagnostic criteria for SLE, diffuse miliary nodules in both lungs were observed on a chest computed tomography scan. A series of tests, including pathological testing of bronchoscopy brushes, T-lymphocyte culture + interferon assay (A + B), tuberculin test, detection of mycobacterium TB DNA, and acid-fast bacilli smear in bronchoalveolar lavage fluid, were unable to confirm the presence of TB infection. We considered the patient to have ALP. After beginning ALP therapy, his symptoms disappeared, and the imaging and hematological results returned to normal. Miliary TB and ALP have similar clinical manifestations and imaging changes, which make diagnosis difficult. This case highlights the need to ensure accurate diagnosis and treatment to improve prognosis.

## 1 Introduction

Systemic lupus erythematosus (SLE) is an autoimmune disease characterized by the production of multiple autoantibodies and involves multiple systems and organs [1]. The incidence of pulmonary involvement in SLE is more common than some connective tissue diseases [2]. Pulmonary involvement is more common in men, particularly in the later stages of the disease, where it is associated with disease prognosis and can increase mortality [3,4]. Pulmonary involvement in SLE can be manifested by chronic interstitial pneumonia, pulmonary fibrosis, pleurisy, diffuse alveolar hemorrhage, pulmonary vascular embolism, and pulmonary hypertension, among others [5]. Even so, acute lupus pneumonitis (ALP), particularly ALP that resembles miliary tuberculosis (TB), is rare.

Miliary TB, an infectious disease caused by the spread of TB through blood, is common in people with low immunity due to long-term use of hormones and immunosuppressants [6,7]. The clinical manifestations of miliary TB and ALP are similar, including fever, cough, night sweats, and shortness of breath. Imaging features show diffuse alveolar infiltration, which lacked specificity [8,9,10]. Clinically, miliary TB and ALP are easily confused and largely indistinguishable.

Here, we present a rare case of ALP mimicking miliary TB that occurred in a 19-year-old male student, which manifested as fever, cough, muscle aches, and joint pain. At the early stage of the disease, uniform miliary nodules were observed on chest computed tomography (CT) image. We further explain the difference between ALP and miliary TB in terms of imaging, clinical manifestations, and laboratory examination results.

## 2 Case presentation

A 19-year-old male student was referred to the Center for Endocrine Metabolism and Immune Diseases, Beijing Luhe Hospital, Capital Medical University, Beijing, for fever that had persisted for 26 days (highest temperature: 39.5℃). The patient had gradually developed symptoms of cough, fatigue, muscle aches, pain in both knee and toe joints, and rashes on the face and scalp, with no evidence of night sweats, hemoptysis, hair loss, dental ulcers, or Raynaud’s phenomenon. He had suffered from hypertension for 3 years. He also had a history of leukopenia for 1.5 years, with the presence of antinuclear antibodies (ANA) 1:160 and anti-β2GP1 IgM (+). However, he did not seek further treatment. He had no notable medical, family, or psycho-social history. On physical examination, swollen, soft, non-tender lymph nodes were felt on both sides of the submandibular, together with thick breathing sounds in both lungs, but no rales. Laboratory examination revealed a white blood cell (WBC) count of 1.64 × 10^9^/L, hemoglobin (Hb) level of 99 g/L, platelet (PLT) level of 56 × 10^9^/L, complement C3 level of 0.206 g/L, and complement C4 level of 0.077 g/L were decreased. The erythrocyte sedimentation rate (ESR) was 90 mm/h, and the C-reactive protein (CRP) level was 10.67 mg/L. Blood gas analysis revealed a pH of 7.480, as well as PaCO_2_ of 32 mmHg, and PaO_2_ of 70 mmHg. The results of tumor marker tests, common virus-related tests, procalcitonin, 1,3-β-d-glucan test (G test), and galactomannan test (GM test) were generally normal. However, we noted positive indicators for autoantibodies, including ANA 1:640 (reference range: <1:40), anti-dsDNA 1:40 (reference range: <1:5), and lupus anticoagulant (LA) 1.76 (reference range: ≦1.2). Additionally, the 24-h urinary protein quantity (24 h-UPro) was 1.34 g/24 h. Color Doppler ultrasound showed that the lymph nodes in the neck, supraclavicular fossa, axilla, and groin area were swollen and well-defined. Bone marrow and peripheral blood smears were conducted to screen for blood system abnormalities, but both results were normal and not indicative of hematological diseases. Based on clinical manifestations and examination results, we considered a diagnosis of SLE.

**Figure 1:**
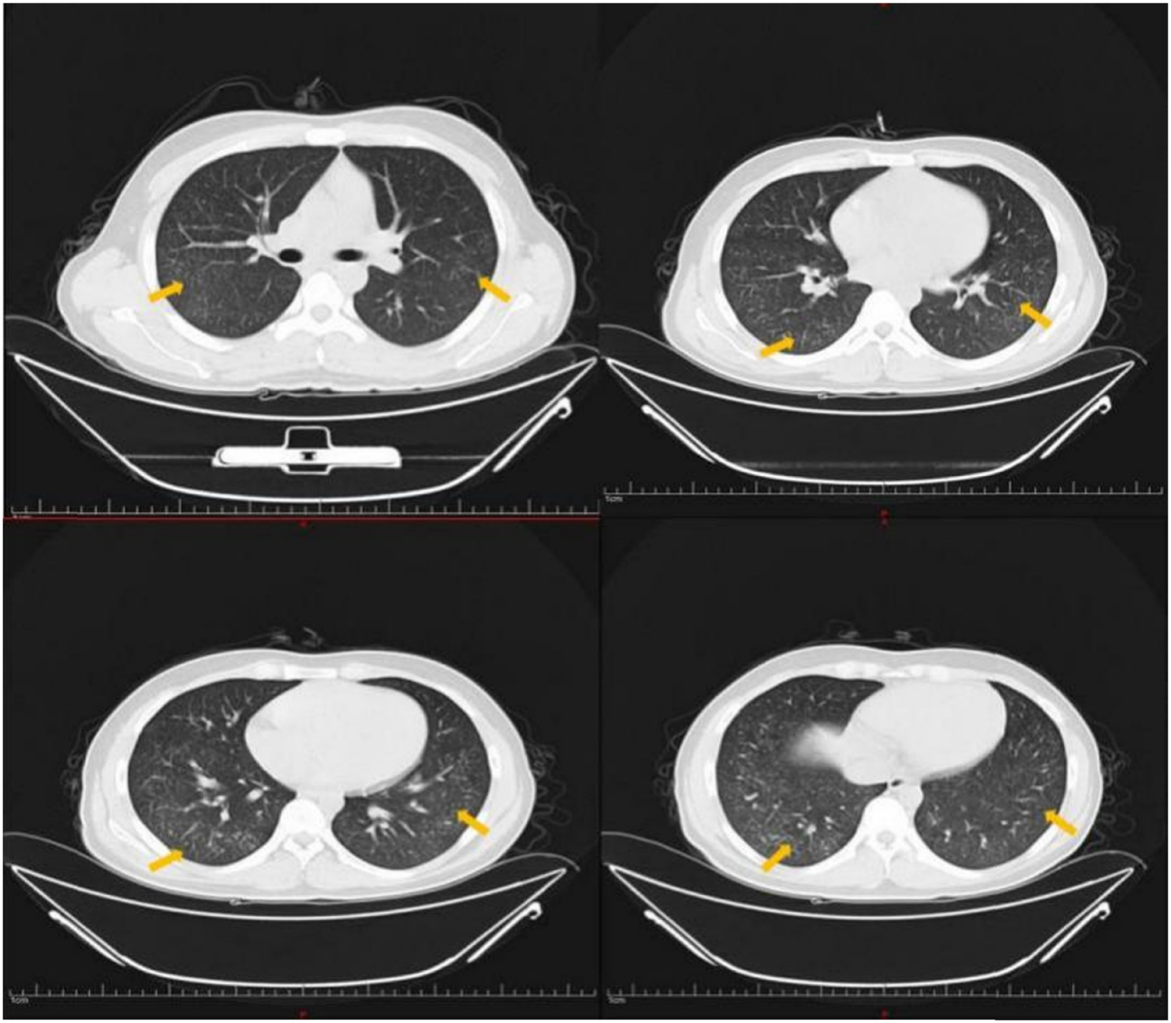
Diffuse miliary nodules in both lungs were observed on chest CT.

**Figure 2:**
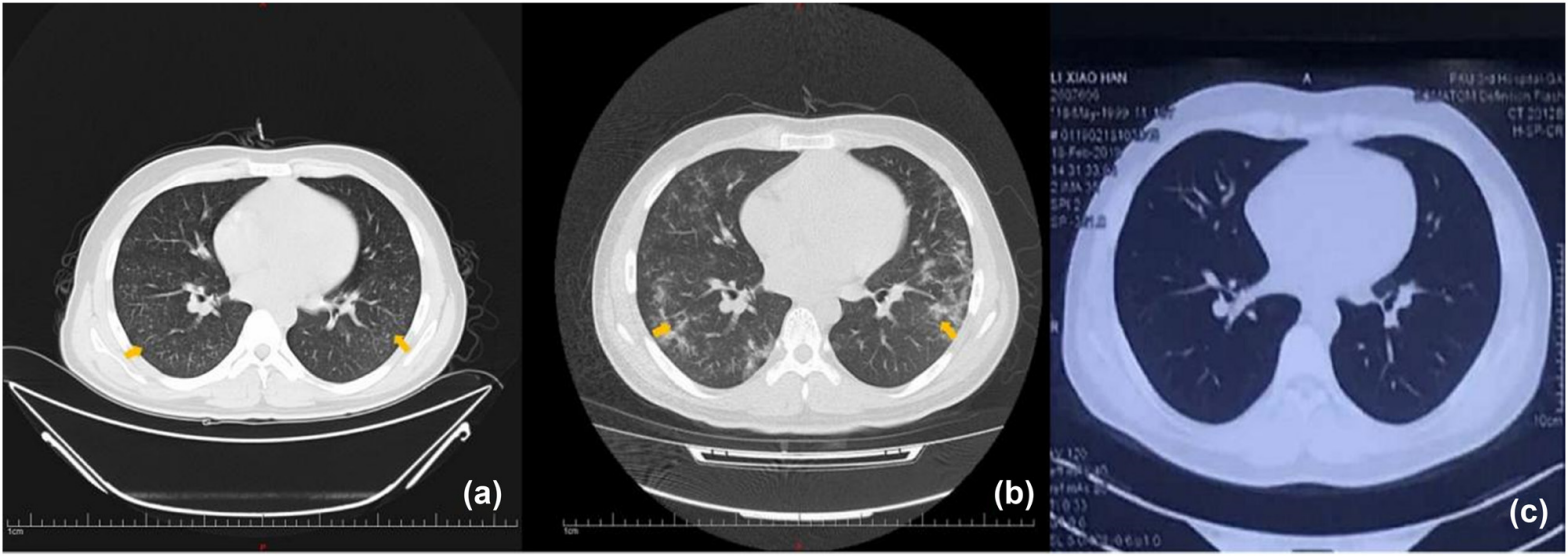
(a) Diffuse miliary nodules were observed in both lungs 14 days after the onset of fever; (b) Patchy shadows in the lower and middle fields of both lungs 31 days after the onset of fever (3 days after empirical treatment; 5 days after admission). (c) After 46 days of treatment, no nodules or shadows were observed in either lung.

**Figure 3:**
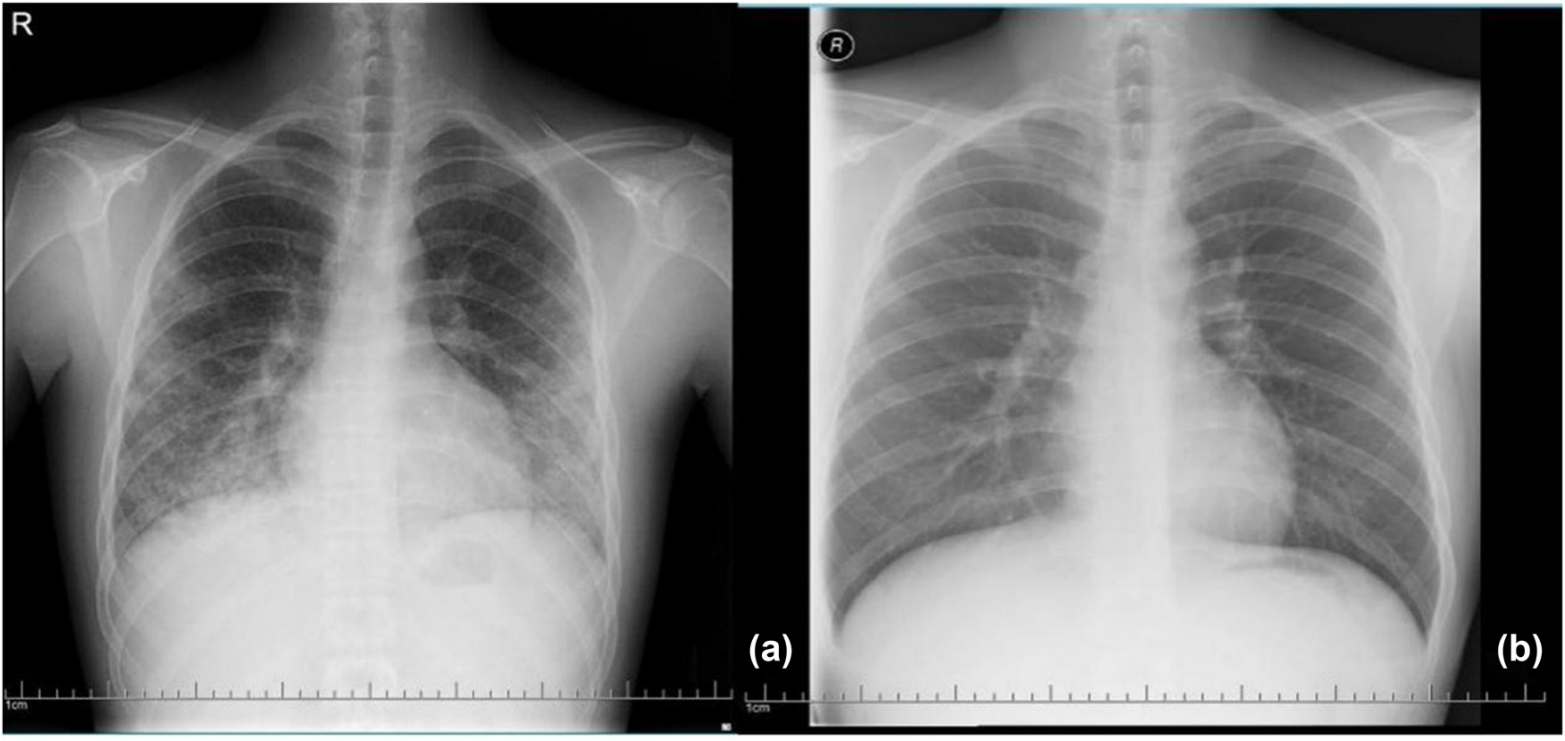
(a) Two days after admission, chest X-ray showed multiple plaques in both lungs. (b) After 5 months of treatment, no high-density shadows were observed on chest X-ray.

However, the results of the patient’s chest CT scan were confusing. Diffuse miliary nodules in both lungs were observed on chest CT after 14 days of fever (Figures 1 and 2a). This led us to consider that the patient had miliary TB. Before being hospitalized at our center, the patient visited many hospitals, but it was unclear whether he had been infected with TB. We completed a series of tests to confirm the presence of mycobacterium TB infection. The pathological results of bronchoscopy brushes of the right lower basal segment identified epithelial cells and lymphocytes, but acid-fast bacillus was not observed. In parallel, the results of T lymphocyte culture + interferon assay (A + B), tuberculin test (PPD), detection of mycobacterium TB DNA, and acid-fast bacilli smear in bronchoalveolar lavage fluid were negative. The results of the pulmonary function test showed moderate mixed ventilation dysfunction and small airway dysfunction. The residual gas volume increased, the total lung volume decreased, and the total residual gas ratio increased. Additionally, both the amount of dispersion and the coefficient of dispersion decreased. Two days after admission, a chest X-ray was completed, which showed multiple plaques in both lungs (Figure 3a). Considering the rapid progression of the patient’s disease in a short period of time, empirical intravenous immunoglobulin (IVIG) and methylprednisolone (MP) therapy were temporarily given. Three days after empirical treatment (31 days after the onset of fever), the patient underwent a repeat chest CT, at which point, patchy shadows were observed in the lower and middle fields of both lungs (Figure 2b).

**Figure 4:**
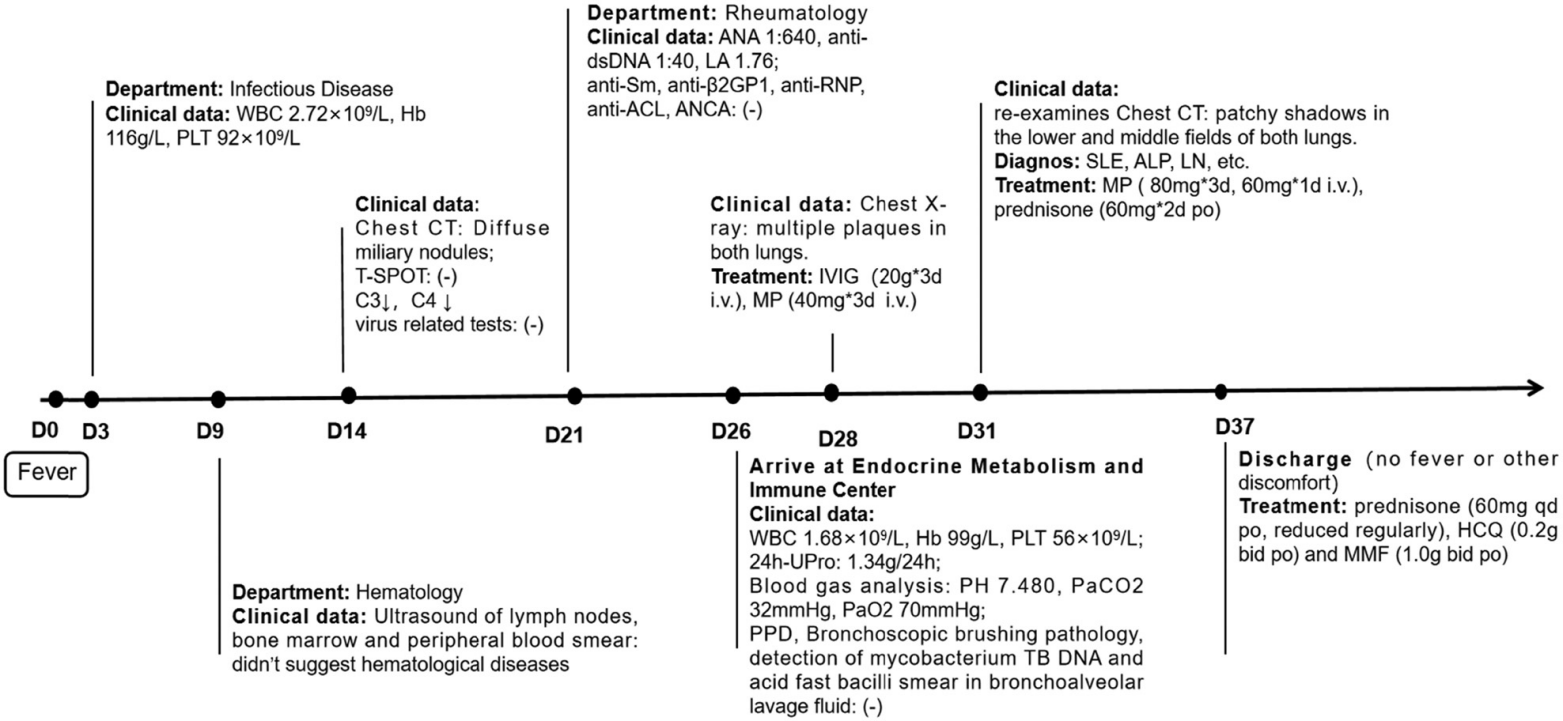
Diagnosis and treatment of a case of acute lupus pneumonia. The first day of fever was recorded as D0. WBC: white blood cells, Hb: hemoglobin, PLT: platelet, C3: complement C3, C4: complement C4, T-SPOT: T-lymphocyte culture + interferon assay (A + B), PPD: tuberculin test, ANA: antinuclear antibodies, LA: lupus anticoagulant, anti-ACL: anti-cardiolipin antibodies, ANCA: neutrophil cytoplasmic antibodies, 24-h-Upro: 24-h urinary protein quantity, SLE: systemic lupus erythematosus, ALP: acute lupus pneumonitis, LN: lupus nephritis, IVIG: intravenous immunoglobulin, MP: methylprednisolone, HCQ: hydroxychloroquine, MMF: mycophenolate mofetil, i.v.: intravenous injection, po: orally, qd: once a day, bid: twice a day.


At present, there is no evidence to support the diagnosis of miliary TB. Therefore, we believe that the miliary changes in both lungs represent a rare imaging manifestation of ALP. The patient continued to receive intravenous MP. After 7 days of treatment, the patient’s temperature returned to normal, and there was no discomfort such as cough and joint pain. In the meantime, the WBC count (5.86 × 10^9^/L) and PLT (154 × 10^9^/L) and ESR 45 mm/h levels recovered. Out of hospital, the patient was given prednisone, hydroxychloroquine, and mycophenolate mofetil (Figure 4). After 46 days of treatment, the miliary nodules and patchy shadows disappeared in chest CT (Figure 2c). After 5 months of treatment, chest X-ray showed no discernible high-density shadows (Figure 3b). Meanwhile, the blood routine examination results, and levels of CRP, ESR, complement C3, and complement C4 returned to normal.

**Informed consent:** Informed consent has been obtained from all individuals included in this study.
**Ethical approval:** The research related to human use has been complied with all the relevant national regulations, institutional policies, and in accordance with the tenets of the Helsinki Declaration and has been approved by the authors’ institutional review board or equivalent committee.

## 3 Discussion

SLE is an autoimmune disease, which presents with vasculitis as the main pathological change. Pulmonary involvement can occur at any stage of SLE and is consistently reported in approximately 50–70% of SLE cases [11]. Pleural involvement is the most common lung manifestation of SLE, with an incidence of 20–90% [3,12]. ALP, a sudden non-infectious pneumonia in patients with SLE, has a low incidence rate of only 1–4% [13,14]. ALP is characterized by fever, cough, dyspnea, hypoxemia, chest pain, and hemoptysis, among others. Bilateral or unilateral patchy consolidation, focal atelectasis, diaphragmatic elevation, or pleural effusion can be seen on ALP imaging [15]. The mortality of ALP is very high (up to 50%); therefore, early diagnosis and treatment are crucial.

TB, caused by the bacillus mycobacterium TB, is one of the top 10 causes of disease-related death worldwide. The World Health Organization’s global TB reports 2019 reported that approximately 10.0 million people were infected with TB globally in 2018, with people in China accounting for 9%. Unfortunately, China is the country with the second largest share of the global TB burden due to the emergence of drug-resistant TB [16,17]. The incidence of TB among patients with SLE is 1.16 per 100 person-years [18]. In patients with SLE, TB infection has not only a high incidence but also a more severe degree of disease, with a fatality rate of 15.6% [19]. Clinical observations show that the clinical manifestations of patients with SLE with TB infection are almost identical to those of simple TB infection and SLE, which makes diagnosis difficult. Several Chinese studies have shown that the incidence of miliary TB in patients with SLE is 35.0%, which represents an important reason for the high fatality rate of SLE [19]. Therefore, clinicians should promptly identify the presence of TB to improve prognosis in this patient population.

In this case, a 19-year-old young boy, who suffered from hyperpyrexia, rashes, leukopenia, thrombocytopenia, urinary protein >0.5 g/24 h, and several types of positive autoantibodies, was definitive diagnosed with SLE, based on 2019 European League Against Rheumatism/American College of Rheumatology Classification Criteria for SLE [20]. However, he also exhibited symptoms of fever, cough, fatigue, and hypoxemia, as well as demonstrating miliary nodules of both lungs that were uniform in size, density, and distribution on chest CT. Therefore, we considered whether the pulmonary manifestation was a component of ALP or SLE with miliary TB. The positive rate of sputum smear, TB antibody test, and PPD test is only 14.3–41.8% in patients with SLE with TB [21]. Therefore, obtaining histopathology and tissue culture results as soon as possible is of great significance to this case. Moreover, the clinical manifestations of patients with SLE with TB infection are non-specific and are often difficult to distinguish from those of SLE itself. The diagnosis of SLE combined with TB is more challenging. In this case, despite attempting to improve the TB test, we found no evidence of TB infection. We have not completely ruled out the possibility of TB infection at this time. We administered the patient IVIG (20 g daily) and MP (40 mg daily). Three days later, the temperature of the patient was normal and the WBC count (2.14 × 10^9^/L) and Hb (101 g/L) and PLT (134 × 10^9^/L) levels were increased. Meanwhile, patchy shadows instead of miliary nodules were been seen in the latest chest radiograph and chest CT. At this time, we considered that the miliary changes in the pre-chest CT may be early imaging manifestations of ALP, rather than miliary TB, and the follow-up results of the patient without anti-TB treatment further verified the correctness of our diagnosis.

The lessons from this rare case of ALP mimicking miliary TB can be summarized as follows. The clinical presentation of ALP is nonspecific. ALP was caused by acute injury of alveoli and capillaries. Patients often develop hypoxemia and hypocapnia. Imaging changes in early ALP with diffuse miliary nodules in both lungs are very rare. At this time, patients may not have serious respiratory tract symptoms, such as dyspnea, cyanosis, and hemoptysis, and may only show discomfort such as cough, fever, and fatigue. it is important to conduct thorough viral, fungal, and bacterial (especially TB) testing. In regard to the identification of ALP with miliary TB, besides perfecting PPD, T-spot, TB antibody, and sputum smear, it is important to actively improve the TB-related pathology and culture inspection. If there is no evidence of TB infection, we can also re-take the chest X-ray or chest CT in a short period of time. The imaging performance of a patient with ALP will change greatly, and scattered patchy shadows may appear gradually, especially in the lower lung field. However, the imaging performance of miliary TB does not change significantly in a short time. In the meantime, if we consider a diagnosis of ALP, we can give 40–80 mg glucocorticoid treatment empirically, while also paying close attention to the changes in clinical manifestations, blood routine, ESR, CRP, blood gas analysis, and chest CT.

We also analyzed two similar published case reports (Table 1) and found that ALP, similar to miliary TB, is most common in adolescents, with fever and cough being notable at the beginning of the disease. Some patients may also present with myalgia and arthralgia. Laboratory tests show that the three systems are reduced, and anti-dsDNA antibodies are positive. Glucocorticoids and immunosuppressants are the standard treatment for ALP, although plasma exchange and gamma globulin may also be used. Unlike this patient, in the other two cases, it was proven that the miliary nodules on chest CT were not miliary TB given the failure of anti-TB treatment. In the cases reported by YP Zhang and YC Huang, TB examinations were not completed, especially the detection of mycobacterium TB by respiratory histopathology and tissue TB DNA. Additionally, the changes in chest X-ray or CT were not observed before treatment with MP or IVIG in the two previously reported cases given that it was not advantageous to describe the changes in the chest imaging of ALP. In this case report, for the first time, we describe the entire process for the development of ALP chest CT. The process occurs in a short time, so it is crucial to observe the changes in chest imaging in time to distinguish ALP from miliary TB.

**Table 1:** Clinical features of ALP

Clinical features	Case 1	Case 2	Case 3
Age	19	15	19
Gender	Female	Female	Male
Fever	Positive	Positive	Positive
Cough	Positive	Positive	Positive
Oral ulcer	Positive	Negative	Negative
Rash	Negative	Negative	Positive
Myodynia	Negative	Positive	Positive
Neck pain	Positive	Negative	Negative
Joint pain	Positive	Negative	Positive
Alopecia	Positive	Negative	Negative
Serous effusion	Positive	Positive	Negative
Proteinuria	Negative	Positive	Positive
Decreased trilineage	Positive	Positive	Positive
Anti-dsDNA antibodies (+)	Positive	Positive	Positive
Anti-Sm antibodies (+)	Positive	Positive	Negative
Complement reduction	Positive	Negative	Positive
IVIG	—	Negative	Positive
Corticosteroids	Positive	Positive	Positive
Immunosuppressor	—	Positive	Positive
Reference	[22]	[10]	—

IVIG: intravenous immunoglobulin.

To sum up, when patients with SLE present with miliary nodules in the lungs, clinicians should take care during the identification process, especially in young patients, and should actively improve the examination of TB, tumor biomarker testing, and other tests for infection with other pathogens, as well as consider the possibility of the disease being complicated with ALP.

## 4 Conclusion

Here, we present a rare case of ALP mimicking miliary TB that occurred in a 19-year-old male student. SLE with miliary TB and ALP have similar clinical manifestations and imaging changes, which often makes them clinically indistinguishable. Every effort should be made to ensure accurate diagnosis and treatment to improve patient prognosis.

## References

[1] Ameer MA, Chaudhry H, Mushtaq J, Khan OS, Babar M, Hashim T, et al. An Overview of Systemic Lupus Erythematosus (SLE) Pathogenesis, Classification, and Management. Cureus. 2022 Oct;14(10):e30330. 10.7759/cureus.30330 PMC9662848 36407159

[2] Yoo H, Hino T, Han J, Franks TJ, Im Y, Hatabu H, et al. Connective tissue disease-related interstitial lung disease (CTD-ILD) and interstitial lung abnormality (ILA): Evolving concept of CT findings, pathology and management. Eur J Radiol Open. 2020 Dec;8:100311. 10.1016/j.ejro.2020.100311 PMC7750149 33364263

[3] Shin JI, Lee KH, Park S, Yang JW, Kim HJ, Song K, et al. Systemic Lupus Erythematosus and Lung Involvement: A Comprehensive Review. J Clin Med. 2022 Nov;11(22):6714. 10.3390/jcm11226714 PMC9698564 36431192

[4] Adwan MH, Qasem U, Mustafa KN. In-hospital mortality in patients with systemic lupus erythematosus: a study from Jordan 2002-2017. Rheumatol Int. 2020 May;40(5):711–7. 10.1007/s00296-020-04538-z 32146489

[5] Amarnani R, Yeoh SA, Denneny EK, Wincup C. Lupus and the Lungs: The Assessment and Management of Pulmonary Manifestations of Systemic Lupus Erythematosus. Front Med (Lausanne). 2021 Jan;7:610257. 10.3389/fmed.2020.610257 PMC7847931 33537331

[6] Pelayo J, Ruddiman K. Miliary Tuberculosis. N Engl J Med. 2020 Sep;383(12):e78. 10.1056/NEJMicm2001934 32937050

[7] Wang EW, Okwesili CN, Doub JB. Tuberculosis the great masquerader. IDCases. 2022 Jun;29:e01541. 10.1016/j.idcr.2022.e01541 PMC9233208 35761798

[8] Elzein F, Elzein A, Mohammed N, Alswailem R. Miliary tuberculosis mimicking systemic lupus erythematosus flare. Respir Med Case Rep. 2018 Sep;25:216–9. 10.1016/j.rmcr.2018.09.005 PMC6143694 30237973

[9] Li JC, Fong W, Wijaya L, Leung YY. Disseminated tuberculosis masquerading as a presentation of systemic lupus erythematosus. Int J Rheum Dis. 2018 Jan;21(1):352–5. 10.1111/1756-185X.13195 28971575

[10] Huang YC, Lin YT, Yang YH, Wang SJ, Yang CM, Chiang BL. Acute lupus pneumonitis mimicking pulmonary tuberculosis: a case report. J Microbiol Immunol Infect. 2001 Jun;34(2):143–6. 11456361

[11] Di Bartolomeo S, Alunno A, Carubbi F. Respiratory Manifestations in Systemic Lupus Erythematosus. Pharmaceuticals (Basel). 2021 Mar;14(3):276. 10.3390/ph14030276 PMC8003168 33803847

[12] Haye Salinas MJ, Caeiro F, Saurit V, Alvarellos A, Wojdyla D, Scherbarth HR, et al. Pleuropulmonary involvement in patients with systemic lupus erythematosus from a Latin American inception cohort (GLADEL). Lupus. 2017 Nov;26(13):1368–77. 10.1177/0961203317699284 28420071

[13] Marte Furment M, Sharma S, Pabolu S. Systemic Lupus Erythematosus Presenting as Acute Lupus Pneumonitis during Pregnancy. Case Rep Rheumatol. 2020 Dec;2020:8839410. 10.1155/2020/8839410 PMC7769658 33414977

[14] Cantero C, Vongthilath R, Plojoux J. Acute lupus pneumonitis as the initial presentation of systemic lupus erythematosus. BMJ Case Rep. 2020 Jul;13(7):e234638. 10.1136/bcr-2020-234638 PMC7342476 32641438

[15] Santamaria-Alza Y, Sanchez-Bautista J, Fajardo-Rivero JE, Figueroa Pineda CL. Acute respiratory involvement in Colombian patients with systemic lupus erythematosus undergoing chest computed tomography. Int J Rheum Dis. 2019 Oct;22(10):1825–31. 10.1111/1756-185X.13686 31496073

[16] World Health Organization. Global tuberculosis report 2019. France: World Health Organization; 2019.

[17] Chaw L, Chien LC, Wong J, Takahashi K, Koh D, Lin RT. Global trends and gaps in research related to latent tuberculosis infection. BMC Public Health. 2020;20(1):352. 10.1186/s12889-020-8419-0 PMC7079542 32183753

[18] Wu Q, Liu Y, Wang W, Zhang Y, Liu K, Chen SH. Incidence and prevalence of tuberculosis in systemic lupus erythematosus patients: A systematic review and meta-analysis. Front Immunol. 2022 Jul;13:938406. 10.3389/fimmu.2022.938406 PMC9355093 35935948

[19] Liu L, Shen M. Pathogenesis and clinical features of tuberculosis in patients with systemic lupus erythematosus. Zhong Hua Yi Xue Za Zhi. 2012;92(41):2949–50.

[20] Aringer M, Costenbader K, Daikh D, Brinks R, Mosca M, Ramsey-Goldman R, et al. European league against rheumatism/american college of rheumatology classification criteria for systemic lupus erythematosus. Arthritis Rheumatol. 2019;71(9):1400–12. 10.1002/art.40930 PMC6827566 31385462

[21] Wang D, Ma L. Literature review of systemic lupus erythematosus associated with tuberculosis infection in China. Zhong Hua Feng Shi Bing Xue Za Zhi. 2009;13:599–602.

[22] Zhang YP, Tang FL. One case of systemic lupus erythematosus misdiagnosed as miliary tuberculosis. Chin J Pract Inter Med. 2001;21(1):57.

